# Identification and treatment of *Enterococcus avium*-induced diabetic foot ulcer: a case report and microbiome analysis

**DOI:** 10.3389/fmed.2024.1502337

**Published:** 2024-12-20

**Authors:** Yuanling Jin, Tao Zhu, Xiao Cai, Zheng Fu, QiangLong Pan, HaiXia Tu, ShouXing Wang, Yan Li

**Affiliations:** ^1^Department of Clinical Laboratory, Sir Run Run Hospital, Nanjing Medical University, Nanjing, China; ^2^Department of Hand Surgery, Sir Run Run Hospital, Nanjing Medical University, Nanjing, China

**Keywords:** diabetic foot ulcers, *E. avium*, microbiome analysis, second-generation sequencing technology, minimal inhibit concentration

## Abstract

**Abstract:**

Diabetic foot ulcer (DFU) is a severe complication of diabetes. Due to conservative or delayed treatment, the majority of DFU patients frequently miss the optimal treatment window, thereby leading to amputation. Despite being a rare pathogen with low virulence, *Enterococcus avium* (*E. avium*) exhibits some antibiotic resistance and can be fatal for immunocompromised patients. This report describes a DFU case, caused by *E. avium* infection due to exposure to poultry. Wound microbiota was dynamically monitored using bacterial culture followed by 16S rRNA gene sequencing throughout the illness. Combination of antibiotics was administered to control the secondary infection.

**Case report:**

A 56-year-old man presented with a two-week history of redness, swelling, heat, pain, and pus discharge from a ruptured wound on his left heel. The patient was diagnosed with osteomyelitis and a Wagner grade 3 diabetic foot ulcer infection, complicated by the soft tissue infection in the left heel. Strain identification and antibiotic susceptibility tests were immediately performed after admission. The patient underwent three debridement procedures at the DFU site. However, we observed recurrent bacterial infections, based on the clinical progression. Second-generation sequencing detected various pathogens. After targeted treatment with Vacuum sealing drainage (VSD) combined with antibiotic bone cement, the patient’s condition stabilised. A skin graft was subsequently performed. Antibiotics were used to control the infection and blood glucose level was controlled throughout the treatment.

**Conclusion:**

Thus, this report provides a comprehensive description of a DFU case, caused by *E. avium.* Antibiotics and surgical measures should be adjusted according to the pathogens responsible for wound infections in DFU patients. It is important to reduce the mortality and prevent irreversible amputations.

## Introduction

Each year, approximately 18.6 million individuals suffer from diabetic foot ulcers (DFUs) (1) globally. DFUs can impair body functions, reduce quality of life, and increase healthcare utilization (2, 3). However, prompt necrotic tissue debridement and proper wound care are frequently overlooked due to inadequate comprehensive examination of ulcers by primary care physicians, and the empirical use of antibiotics. Additionally, poor glycemic control exacerbates the progression of foot ulcers into soft tissue infections, gangrene, and even limb amputation (4). Approximately half of the DFU patients suffer from peripheral artery disease of lower extremities (5). Fifty percent of ulcers develop an infection; of these, 20% require hospitalization, and 15–20% of moderate-to-severe infections result in limb amputation (3, 6, 7). DFU patients have a five-year mortality rate of 30%, and the mortality rate exceeds 70% in cases of amputation above the ankle (8).

DFUs are one of the leading causes of non-traumatic amputations (9–12). Clinical wounds frequently harbor numerous bacteria and fungi, including *Staphylococcus* and *Streptococcus* species (13). To prevent the progression of infection, empirical antimicrobial medication is necessary before the return of bacterial identification results (14). Diabetic foot infections have been treated with various antibiotic regimens; however, most DFU patients exhibit peripheral vascular disease or diabetic neuropathy with compromised immune functions (15, 16). Consequently, antibiotics cannot penetrate deeply and achieve effective antimicrobial action. This can rapidly induce bacterial resistance (17). Additionally, conventional swab culture methods are ineffective in capturing deep tissue flora, leading to frequent failure of deep infection diagnosis (18, 19).

*Enterococcus avium*, formerly designated as *Group Q Streptococcus*, was named due to its frequent isolation from chicken feces (20), and subsequent findings revealed its presence in feces of all mammals (21). It belongs to the Enterococcus genus and has been an underrecognized human pathogen.

The rarity and diagnostic challenges associated with *E. avium* have historically relegated it to a secondary position behind more prevalent pathogens of the *Enterococcus* genus, such as *E. faecalis* and *E. faecium* (22). However, advancements in genetic testing technologies, which have become globally accessible in the past decade, have led to an increase in reported cases of *E. avium* infections (23, 24). Recent analyses of case reports indicate that *E. avium* can cause various conditions, often complicated by bacteraemia (25, 26), including idiopathic granulomatous mastitis with superimposed *E. avium* infection (27),peritonitis (28–30), biliary tract infections (31), and brain abscesses (32, 33). *E. avium*, a zoonotic bacterium, colonizes multiple body sites and is associated with severe nosocomial infections in humans (33, 34). A recent population-based retrospective study in Region Skåne, Sweden, identified a vancomycin-resistant *E. avium* isolate from a patient with bacteraemia, emphasizing its relevance in clinical settings (26). Conversely, a two-year prospective study conducted at the Medical College, Kolkata, India, found eight *E. avium* isolates, none exhibiting vancomycin resistance (35). Additionally, an intermediate resistance to minocycline was documented in *E. avium* isolated from a 61-year-old Japanese patient with colorectal adenocarcinoma (29). Research from the Medical College, Dammam, Saudi Arabia, identified an *E. avium* strain possessing 14 genes associated with antimicrobial resistance mechanisms, including efflux pumps, suggesting a potential rise in resistance within healthcare environments. These findings underline growing concerns regarding *E. avium*’s resistance and its implications in healthcare settings (36).

Moreover, DFUs have a larger microbial diversity than previously determined by culture-based methods. However, limited information is available on the complexity and dynamic changes of the diabetic foot microbiota; different microorganisms can significantly impact the prognosis of DFU (37). Therefore, this case report aimed to highlight the local microbiota changes and antibiotic sensitivity in a DFU case, caused by rare pathogens.

## Case presentation

### The case

A 56-year-old male patient with a history of type 2 diabetes and liver cirrhosis initially presented with skin redness, swelling, heat, pain, and pus discharge from a ruptured wound on his left heel. His symptoms persisted despite receiving a 1-week course of cefuroxime from a primary care physician at a local hospital. The patient reported no headache, dizziness, nausea, or vomiting after hospital admission. He had a normal appetite, slept well, and had regular urination without any diarrhea. He did not have chills, fever, abdominal distension or pain, cough, sputum production, or recent weight loss.

### Physical examination

Laboratory results showed 6.31 × 10^9^/L white blood cells, 18.1% lymphocytes, 3.27 × 10^12^/L red blood cells, 103 g/L hemoglobin, and 116 × 10^9^/L platelets. Moreover, high-sensitivity C-reactive protein was abnormally elevated at 38.20 mg/L, while the total protein and albumin levels were abnormally down-regulated at 64.6 g/L and 25.2 g/L, respectively. In liver function tests, aspartate aminotransferase and alanine aminotransferase levels were 13 U/L and 8 U/L, respectively. Additional laboratory values were 36 U/L for creatine kinase, 134 mmol/L for sodium, 1.85 mmol/L for calcium, 0.82 mmol/L of phosphorus, 48 μmol/L for creatinine, 141 μmol/L for uric acid, 14.99 mmol/L for glucose, and 0.71 mmol/L for high-density lipoprotein.

**Figure 1:**
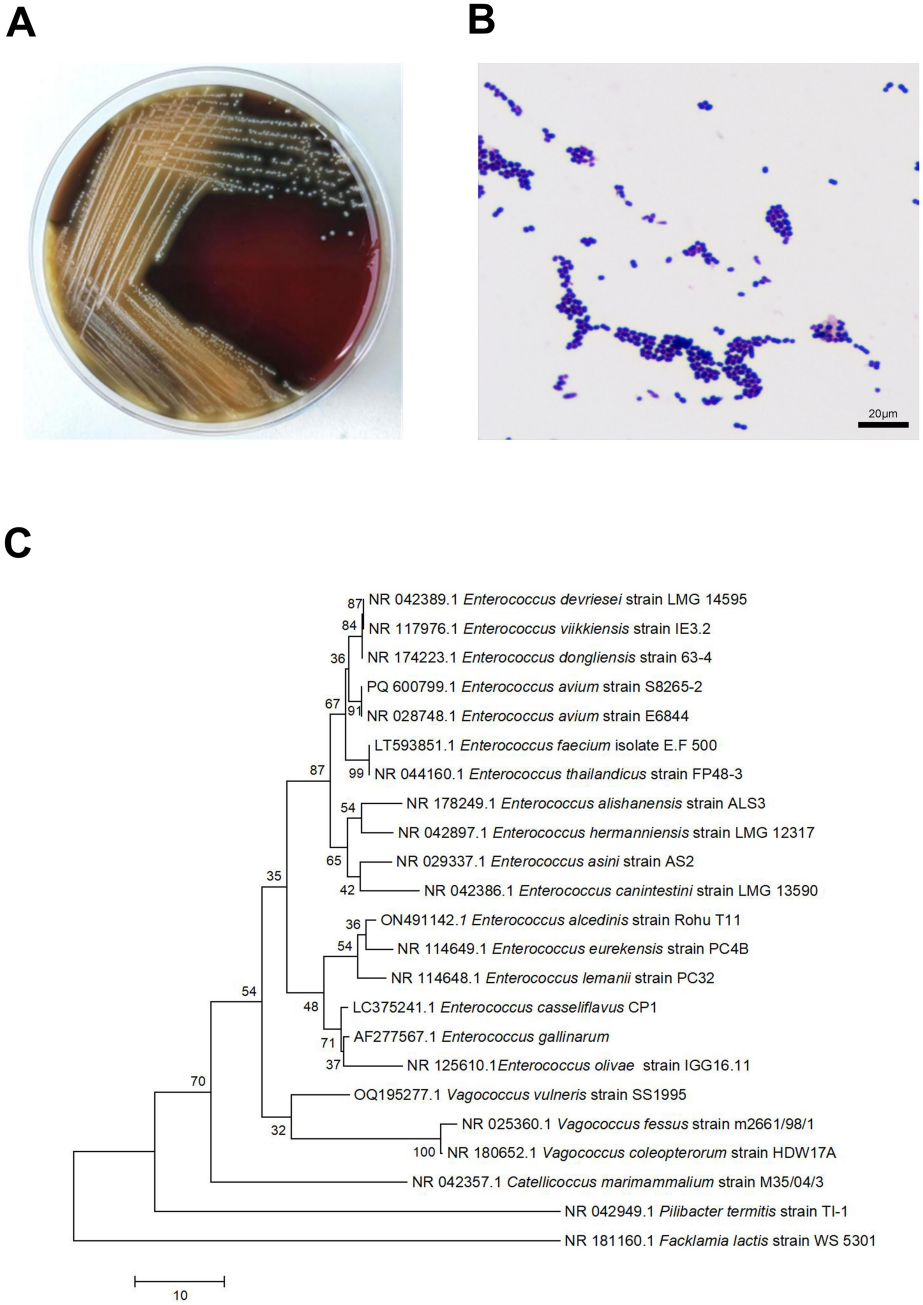
Small, grey, colonies of *Enterococcus avium* were observed on sheep blood agar **(A)**. Microscopic examination of a Gram-stained smear revealed small Gram-positive cocci at 1,000× magnification **(B)**. Neighbor-joining Phylogenetic trees constructed by using 16S rRNA sequences of 1 *E. avium* strains from patients in Hospital. Facklamia lactis strain WS 5301 as the root **(C)**.

After culturing the wound swab, the resultant colonies on Columbia agar plates were pale yellow, smooth, moist, sticky, and 1–2 mm in size, with well-defined edges. The smear’s Gram staining revealed Gram-positive cocci. With a 99% confidence level, the microbial identification instruments (VITEK ® 2 Compact system, Mérieux) identified the isolate as *E. avium*. The patient reported a past exposure to poultry. The pathogenic colonies were in a concentration of up to 50,000 colony-forming units (CFU)/mL, indicating their significance in this infection. For 16S rRNA sequencing, pure colonies were supplied to Sangon Bio Co., Ltd. Phylogenetic tree comparisons of sequencing results analysed by *E. avium* (PQ600799.1) using the Basic Local Comparison Search tool^1^ showed 91% homology to *E. avium* (Figure 1).

## Diagnostic assessment

The patient in this case report was assessed and diagnosed using the Meggitt-Wagner classification system. Examination of the left heel revealed trauma measuring approximately 3 cm × 2 cm on the lateral side and 2 cm × 2 cm on the posterior aspect, with a depth reaching the fascia and subcutaneous tissue exposure. Minimal pus secretion was observed, along with fluctuating pain and pressure sensations in the surrounding skin. Notable signs included putrefaction and crust formation within the wound, redness, and swelling around the trauma site and dorsum of the foot, with the affected area exhibiting elevated skin temperature compared to the contralateral side. Pressure-induced pain and a malodorous discharge were also present. Despite these findings, muscle strength and sensation in the left lower limb were intact, though hypesthesia was noted on the dorsum of the foot. MRI findings indicated bone marrow oedema in the left heel bone and soft tissue oedema surrounding the heel bone and plantar fascia. Based on these clinical and imaging findings, the diabetic foot ulcer (DFU) was classified as Wagner grade 3. Wound microbiological cultures confirmed an extensive infection of the diabetic foot caused by *E. avium*.

## Treatment

Following 6 days of anti-inflammatory therapy, the patient underwent necrotic infection site debridement on the left heel and negative pressure wound therapy (VSD).

The patient underwent a second debridement procedure on the left lower limb under nerve block anesthesia on the 15th day after admission. A vacuum-sealed drainage device was subsequently applied to the wound. Fresh granulation tissue was observed covering the wound during the procedure; however, microbial colonies and purulent exudate were visible on the calcaneus bone’s surface. After removing the remaining necrotic tissue and cleaning the wound with saline followed by hydrogen peroxide, the surgeon placed a vacuum-sealed drainage device. The wound received pressure dressing with sterile surgical cotton and elastic bandages. After the wound swabs were sent for culture, *Staphylococcus aureus* and *Escherichia coli* strains were isolated on the 15th, 20th, and 24th days. All strains showed similar antibiotic resistance patterns. *Enterococcus faecium* was also isolated on the 25th day.

The patient’s left foot wound displayed extensive infection of necrotic tissue and foul odor on the 31st day after admission. Subsequently, he underwent an extensive debridement of the left foot infection, necrotic tissue removal, and vancomycin-impregnated bone cement application. Both the 15- and 31-day debrided necrotic tissues were sent for 16S rRNA sequencing for microbial classification.

**Figure 2:**
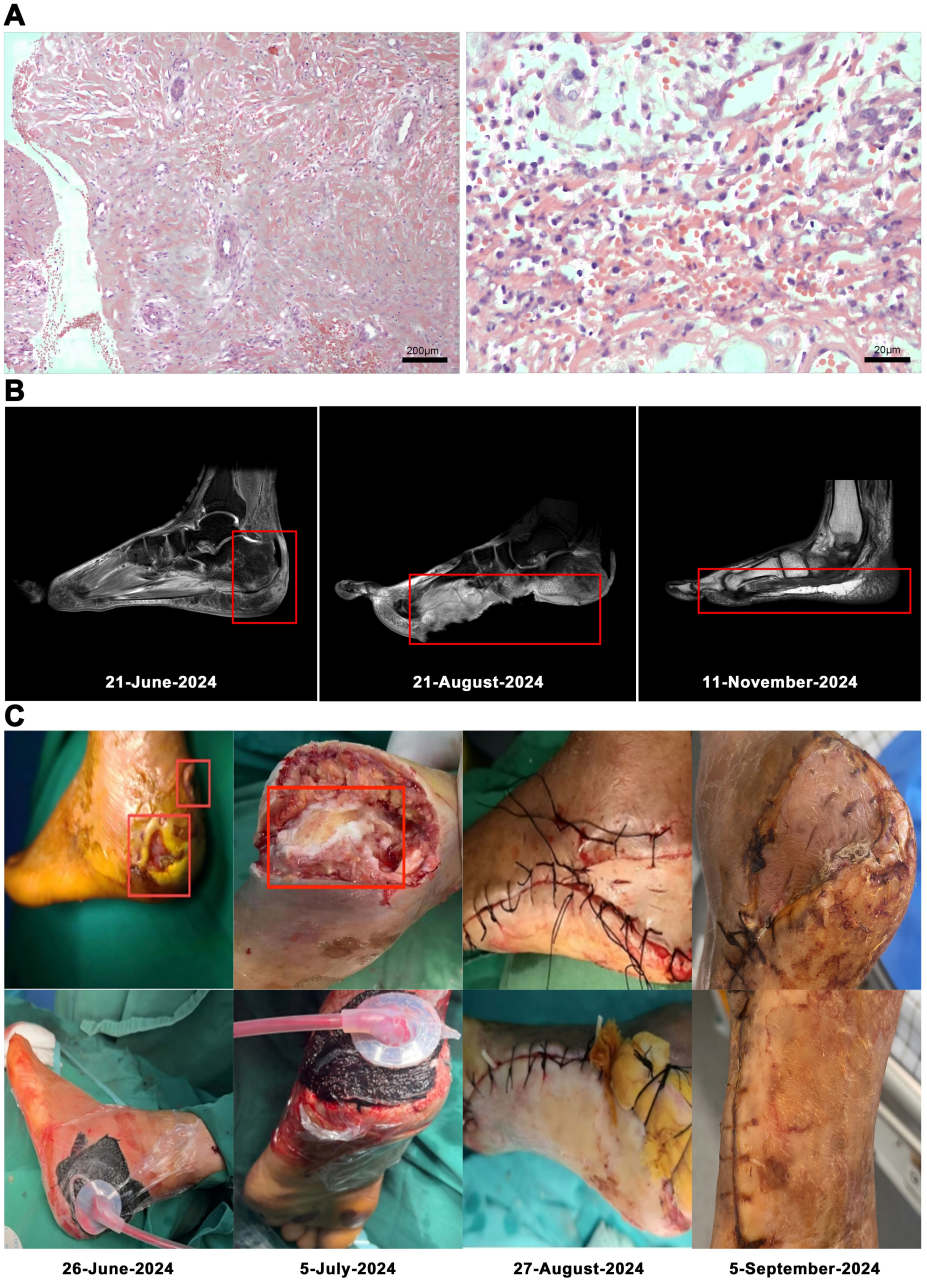
Low-power magnification of left heel infected necrotic tissue focal subcutaneous hemorrhagic necrosis with acute and chronic inflammation (hematoxylin and eosin stain, original magnification × 100). Histopathologic examination at high magnification showed lymphocytic and plasma cell infiltration (hematoxylin and eosin stain, original magnification × 1,000; **A**). MRI: foot At initial presentation; Status before foot transplantation; After foot transplantation, follow-up status **(B)**. The patient’s surgery photos **(C)**.

On the 53rd day, no bacterial growth was observed. The patient underwent left foot debridement and split-thickness skin grafting under epidural anesthesia. Following the complete excision of old skin edges and necrotic tissue, the wound was thoroughly irrigated by a pulsatile lavage system. Approximately the size of the left heel defect, a split-thickness skin graft was harvested from the lateral aspect of the left calf and was sterilely wrapped. Hemostasis and adhesion prevention were achieved using absorbable hemostat and a 3 mL adhesion prevention agent, respectively. The patient’s magnetic resonance imaging (MRI), postoperative tissue pathology results and surgery photos are shown in (Figure 2).

By the 62nd hospitalization day, the physician confirmed that the skin had covered the wound completely, with good postoperative recovery. The wound healed completely till the 71st day. Subsequently, the patient exhibited symptoms of walking with a slight limp at the first follow-up visit after completing treatment and being discharged from the hospital. Upon examination, the foot wound had fully healed, with no evidence of redness, swelling, oozing, necrotic tissue, or other abnormalities. Additionally, there were no apparent signs of infection recurrence.

## Microbiome analysis

**Figure 3:**
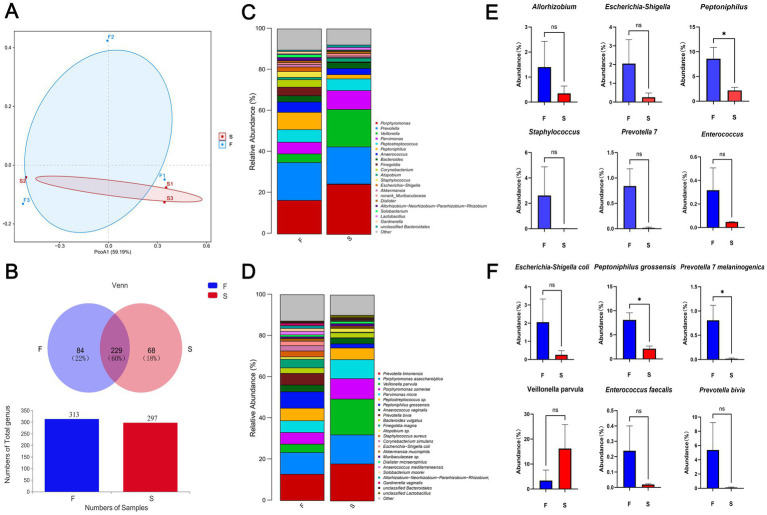
Beta diversity indicators PcoA **(A)** and Venn **(B)** between groups F and S; histogram of differences in relative abundance at the genus level **(C)**; histogram of differences in relative abundance at the species level **(D)**; Genus and species data are provided as mean earth SD and were analyzed by Welch’s t-test comparison test **(E,F)**. *p < 0.05; **p < 0.01.

**Figure 4:**
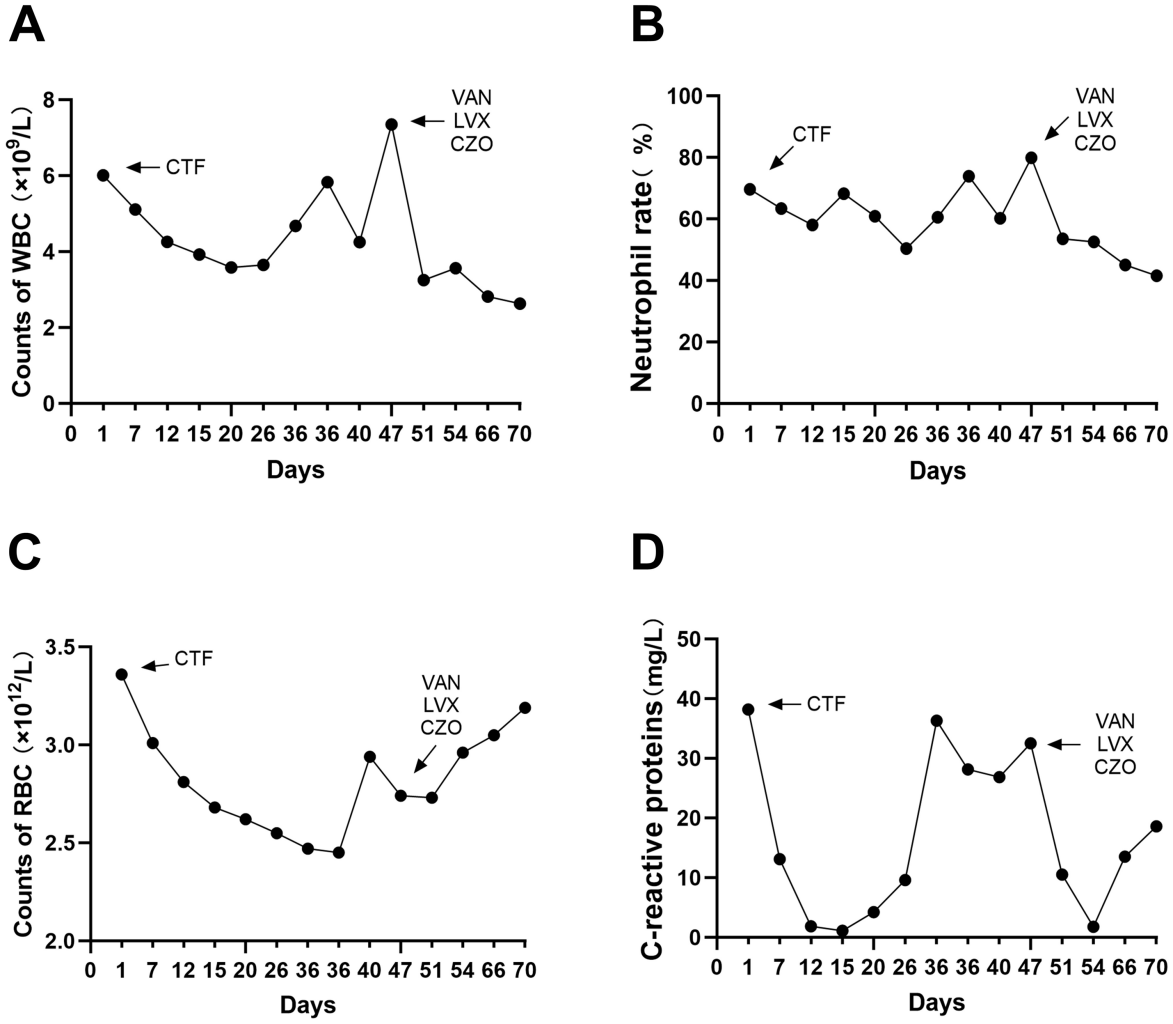
Blood routine and C-reactive protein indexes were detected in hosipitalization, respectively **(A–D)**.

Upon admission, the patient showed enhanced high-sensitivity C-reactive protein and glucose levels as well as reduced total protein, albumin, and red blood cell levels. Hence, human albumin was administered intravenously to correct these abnormalities. Moreover, an insulin glargine and humalog regime helped stabilize blood glucose levels. On the 15th day of ceftriaxone empirical treatment (F), the wound cultures yielded *S. aureus*, *E. coli*, and *E. faecium*. After consulting the microbiology department, appropriate antibiotic therapy was implemented, using a combination of cefazolin, levofloxacin, and vancomycin based on the sensitivity data (Table 1). A second debridement was performed on the 31st day (S). In order to further investigate the alterations in the local microbial spectrum of DFU, high-throughput sequencing of microbial genes was done to analyze changes in microbial communities between the two debridement sessions (Figure 3). Principal coordinate analysis (PcoA) at the OTU level indicated that the two groups’ microbial community structure and composition were similar. The Venn diagram results showed 229 shared genera, with 84 and 68 genera being unique to the F and S groups, respectively. The top 10 genera were *Porphyromonas*, *Prevotella*, *Veillonella*, *Parvimonas*, *Peptostreptococcus*, *Peptoniphilus*, *Anaerococcus*, *Bacteroides*, *Finegoldia*, and *Corynebacterium*. The top 10 species included *Prevotella timonensis*, *Porphyromonas asaccharolytica*, *Veillonella parvula*, *Porphyromonas somerae*, *Parvimonas micra*, *Peptostreptococcus* sp., *Peptoniphilus grossensis*, *Anaerococcus vaginalis*, *Prevotella bivia*, and *Bacteroides vulgatus*. At the genus level, the F group had a substantially larger proportion of *Peptoniphilus* than the S group (*p* < 0.05). However, *Allorhizobium*, *Escherichia-Shigella*, *Staphylococcus*, *Prevotella 7 melaninogenica*, and *Enterococcus* were more prevalent in the F group. At the species level, the F group had substantially larger proportions of *P. grossensis* and *P. 7 melaninogenica* (*p* < 0.05). Moreover, the F group also displayed significantly larger proportions of *Escherichia-Shigella coli*, *S. aureus*, *P. bivia*, and *Enterococcus faecalis*. After adjusted antibiotic use on day 47, hematological indicators showed significantly reduced white blood cell and neutrophil ratios; however, decreased C-reactive protein level was not statistically significant. Additionally, red blood cell levels increased significantly (Figure 4). After antibiotic therapy adjustments, the patient experienced reduced pain at the left heel wound and had significant improvement in infection symptoms.

**Table 1:** Antibiotic resistance in bacterial strains isolated from wound samples.

Antimicrobial	Gram-positive bacteria						Gram-negative bacteria	
	*Enterococcus avium*		*Enterococcus faecalis*		*Staphylococcus aureus*		*Escherichia coli*	
	MIC (μg/ml)	Interpretaation	MIC (μg/ml)	Interpretaation	MIC (μg/ml)	Interpretaation	MIC (μg/ml)	Interpretaation
Cefoxitin Sodium	2	R						
Ceftaroline Fosamil	≤0.12	R						
Clindamycin	1	R			0.25	R		
Erythromycin			≥8	R	≥8	R		
Gentamicin	≤2	R						
Penicillin					≥0.5	R		
Tetracycline	8	I						
Levofloxacin	4	I	≥8	R				
Cephalothin							16	I

S, susceptible; I, intermediate; R, resistant.

## Discussion

*E. avium* is a Gram-positive, catalase-negative member of the Enterococcus genus and causes approximately 1% of human infections (38). Due to its ability to adapt to harsh environments, it displays extended survival time and the potential to cause nosocomial infections. This case report described a 56-year-old male patient with type 2 diabetes and liver cirrhosis. However, traditional methods, like swab cultures, frequently fail to capture pathogens from deeper wound layers. Since bacterial culture techniques are limited by their ability to cultivate anaerobes and their identification time, which can delay appropriate treatments. Moreover, these methods cannot accurately assess the infection’s extent, depth, severity, or the presence of specific pathogens. Empirical antibiotic therapy does not affect all bacterial species (39). An emerging pathogen diagnostic technique, microbial classification sequencing allows the direct extraction of all microbial DNA from clinical specimens without any culture. This approach enables rapid and accurate identification of pathogens, including rare and opportunistic bacteria, thus facilitating dynamic microbial community monitoring and development of personalized treatment strategies. In this case, microbial classification sequencing revealed significant microbial diversity in DFUs, with anaerobes from mixed communities, including *P. timonensis*, *P. asaccharolytica*, *V. parvula*, *P. somerae*, *P. micra*, *Peptostreptococcus* sp., *P. grossensis*, *A. vaginalis*, *P. bivia*, and *B vulgatus*. This enhanced proportion is associated with deeper diabetic wounds and poorer outcomes. Notably, the proportion of *V. parvula* increased after combined antibiotic therapy. This pathogenic species causes endocarditis (40), meningitis (41), and osteomyelitis (42). Conversely, commonly implicated pathogens in nosocomial infections, including *Escherichia coli*, *Staphylococcus aureus* and *Enterococcus faecalis*, were detected at low levels. This highlights the complexities of wound microbiota and its impact on chronic wound progression. Additionally, predicting microbial community functions and their impact on DFU outcomes might improve future clinical treatment and prognosis. These findings underscore the importance of advanced microbial diagnostics in managing and predicting DFU infections.

DFU is a severe microvascular complication that significantly increases morbidity and mortality rates, respectively. DFU also represents a complex interplay between neuropathy, peripheral arterial disease, foot deformities, and infection. Initiating as superficial cellulitis, the pathogenic microbiota can cause osteomyelitis and gangrenous limb amputations (43). Hence, proper medications can effectively manage these infections. Infections by multiple drug-resistant microorganisms often require susceptibility results-based combination antibiotic therapies (44, 45). Biofilm formation on DFU hinders antibiotic penetration into the wound’s deeper layers, thereby causing antibiotic resistance. Therefore, debridement is an essential and effective step for removing bacterial biofilms in DFU cases. Depending on the wound’s severity, debridement can range from minor procedures to more invasive approaches, including excision, revascularization, or amputation (46). However, intravenous antibiotics do not achieve effective bactericidal concentrations within the wound due to the compromised blood flow to the foot (47). Thus, bone cement, which releases antibiotics slowly and continuously, is effective in maintaining higher local antibiotic concentrations, compared to systemic applications, with an efficiency of 81% in preventing resistant strains (48). Localized drug release minimizes the patient’s systemic circulation exposure, thereby reducing the side effects of antibiotics (49), promoting wound healing, and lowering treatment expenditure. This study has several limitations, including a small sample size, which may have reduced the statistical power for certain comparisons, thereby limiting the generalizability of the findings to the broader population. Future research should address this by incorporating larger sample sizes. Additionally, some bacteria identified may represent contaminants or colonizers rather than pathogens actively contributing to the pathophysiology of diabetic foot infections. Delays in the timely processing of biopsy material may also have influenced the accuracy of culture results.

## Conclusion

As the first documented case of *E. avium* infection in a diabetic patient in China, early detection of *E. avium* played a crucial role in confirming the diagnosis and serves as a significant reference point for guiding future treatment strategies. Microbiology laboratories should utilize several diagnostic techniques like bacterial culture and identification, mass spectrometry, and microbial genomic sequencing to rapidly and accurately identify pathogenic microorganisms (50). Moreover, the prompt delivery of susceptibility reports enables clinicians to adjust antibiotic treatments accordingly. This prevents further deterioration of the patient’s condition, minimizes hospital stays, and reduces the patient’s monetary burden. Dynamic microbiome analysis also provides useful insights for an early clinical diagnosis and infection prognosis by accurately detecting various pathogenic microorganisms.

## Data Availability

The original contributions presented in the study are included in the article, further inquiries can be directed to the corresponding authors.

## Footnotes

^1^ https://blast.ncbi.nlm.nih.gov/Blast.cgi
