# Supplementary material for: Identification and treatment of Enterococcus avium-induced diabetic foot ulcer: a case report and microbiome analysis
Source: Front Med (Lausanne). 2024 Dec 20;11:1502337. doi: 10.3389/fmed.2024.1502337 (PMC11695312; doi:10.3389/fmed.2024.1502337)
Supplement: Supplementary file 1 [file Data_Sheet_1.docx]

**Supplementary Materials**

**Identification and Treatment of Enterococcus avium-Induced Diabetic Foot Ulcer: A Case Report and Microbiome Analysis**

**Yuanling Jin^1†^, Tao Zhu^1†^, Xiao Cai^1†^, Zheng Fu^2^, QiangLong Pan^1^, HaiXia Tu^1^, ShouXing Wang^1*^ and Yan Li^1*^**

^1^ Department of Clinical Laboratory, Sir Run Run Hospital, Nanjing Medical University, Nanjing, China.

^2^ Department of Hand Surgery, Sir Run Run Hospital, Nanjing Medical University, Nanjing, China.

†These authors share first authorship

***, Correspondence:**

Corresponding Authors

[wshx1120@126.com](file:///C:\Users\25585\Desktop\鸟proof\wshx1120@126.com) OR [yanli@njmu.edu.cn](file:///C:\Users\25585\Desktop\鸟proof\yanli@njmu.edu.cn)

**Supplementary Table S1. Primers used for PCR ampliﬁcations.**

| **No.** | **Target Gene** | **Primer Sequence (5’-3’)** | **Annealing**  **Temperature** | **Length (bp)** | **GenBank Reference**  **Sequence** |
| --- | --- | --- | --- | --- | --- |
| 1 | *E.avium* strain E6844 16S ribosomal RNA | CCGTAGAGTTTGATCCTGGCTCAGG/GACGGCTACCTTGTTACGACTTCAC | 57^◦^C | 1524 bp | [NR_028748](https://www.ncbi.nlm.nih.gov/nuccore/NR_028748.1) |
| 2 | *E.avium* strain IRMC1622a 16S ribosomal RNA | AGAGTTTGATCCTGGCTCAG/TACGGCTACCTTGTTACGACTT | 50^◦^C | - | - |
| 3 | *E.avium* strain 352 16S ribosomal RNA | AGAGTTTGATCCTGGCTCAG/  GGTTACCTTGTTACGACTT | 50^◦^C | 1465 bp | [NZ_CP034169](https://www.ncbi.nlm.nih.gov/nuccore/NZ_CP034169.1) |
| 4 | E.avium strain S8265-2 16S ribosomal RNA | GGCTCAGGACGAACGCTGG/ACGACTTCACCCCAATCATCTATCCC | 57^◦^C | 1495bp | [PQ600799](https://www.ncbi.nlm.nih.gov/nuccore/PQ600799) |

**Supplementary Figure S1. PCR amplification of the genes**

| 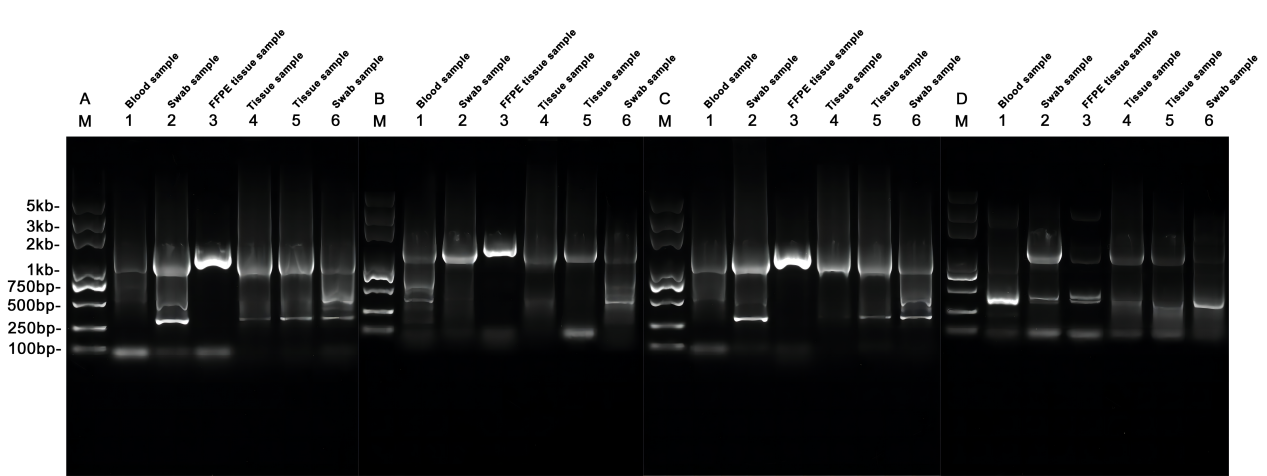 |
| --- |
| **Fig S1.**PCR amplification of (A) 352 (B) E6844 (C) IRMC1622a and (D) S8265-2 genes. (M) Molecular size markers, (1) 26-June Blood sample, (2) 29-June Swab sample, (3) 8-July FFPE (Formalin Fixed Paraffin Embedded) tissue sample, (4)13-July Tissue sample, (5)26-July Tissue sample, (6) 3-September Swab sample. |

**Supplementary Table S2. Laboratory results of patient follow-up**

| **Hematology** |  |
| --- | --- |
| White blood cells | 2.71×10^9^/L |
| Neutrophils | 38.0% |
| Lymphocytes | 48.7% |
| Eosinophils | 2.2% |
| Red blood cells | 3.94×10^12^/L |
| Hemoglobin | 93 g/L |
| Hematocrit | 30.1% |
| Platelets | 104×10^9^/L |
| **Blood chemistry** |  |
| Total protein | 65.0 g/L |
| Albumin | 37.9 g/L |
| Aspartate aminotransferase | 22 U/L |
| Alanine aminotransferase | 13 U/L |
| Sodium | 140 mmol/L |
| Potassium | 4.49 mmol/L |
| Chloride | 105.7 mmol/L |
| Blood urea nitrogen | 7.4 mmol/L |
| Creatinine | 80 umol/L |
| hypersensitive C-reactive protein | 5.29 mg/L |
| Total bilirubin | 11.5 umol/L |
| **Urinalysis** |  |
| GLU | 4+ |
| WBC | 0-1 cells/HPF |
| RBC | 0 cells/HPF |
